# Probing hyper-negatively supercoiled mini-circles with nucleases and DNA binding proteins

**DOI:** 10.1371/journal.pone.0202138

**Published:** 2018-08-16

**Authors:** Carole Saintomé, Emmanuelle Delagoutte

**Affiliations:** 1 Structure des Acides Nucléiques, Télomères et Évolution, Muséum national d'Histoire naturelle, Institut National de la Santé et de la Recherche Médicale, Centre National de Recherche Scientifique, Paris, France; 2 France Sorbonne Université, UFR-927, Paris, France; 3 Physiologie et Génomique de l’Adaptation, Muséum national d'Histoire naturelle, Institut National de la Santé et de la Recherche Médicale, Centre National de Recherche Scientifique, Paris, France; Universite Laval, CANADA

## Abstract

It is well accepted that the introduction of negative supercoils locally unwinds the DNA double helix, influencing thus the activity of proteins. Despite the use of recent methods of molecular dynamics simulations to model the DNA supercoiling-induced DNA deformation, the precise extent and location of unpaired bases induced by the negative supercoiling have never been investigated at the nucleotide level. Our goals in this study were to use radiolabeled double-stranded DNA mini-circles (dsMCs) to locate the unpaired bases on dsMCs whose topology ranged from relaxed to hyper-negatively supercoiled states, and to characterize the binding of proteins involved in the DNA metabolism. Our results show that the Nuclease SI is nearly ten times more active on hyper-negatively supercoiled than relaxed DNA. The structural changes responsible for this stimulation of activity were mapped for the first time with a base pair resolution and shown to be subtle and distributed along the entire sequence. As divalent cations modify the DNA topology, our binding studies were conducted with or without magnesium. Without magnesium, the dsMCs topoisomers mostly differ by their twist. Under these conditions, the *Escherichia coli* topoisomerase I weakly binds relaxed dsMCs and exhibits a stronger binding on negatively and hyper-negatively supercoiled dsMCs than relaxed dsMCs, with no significant difference in the binding activity among the supercoiled topoisomers. For the human replication protein A (hRPA), the more negatively supercoiled is the DNA, the better the binding, illustrating the twist-dependent binding activity for this protein. The presence of magnesium permits the dsMCs to writhe upon introduction of negative supercoiling and greatly modifies the binding properties of the hRPA and *Escherichia coli* SSB on dsMCs, indicating a magnesium-dependent DNA binding behavior. Finally, our experiments that probe the topology of the DNA in the hRPA-dsMC complexes show that naked and hRPA-bound dsMCs have the same topology.

## Introduction

The activity of proteins involved in the DNA metabolism such as specific DNA binding proteins, nucleases or RNA polymerases, is influenced by the topology of the DNA and more specifically by the degree of superhelicity [1–12]. For instance, it was early recognized that the single-stranded DNA binding (SSB) protein of bacteriophage T4 invades less efficiently covalently closed circular than linear double-stranded DNA, due to the strain induced by the strand separation in closed circular DNA [13]. In the mid seventies, Wang monitored the transcriptional activity of the RNA polymerase on DNA samples containing varying numbers of superhelical turns and reported the influence of the level of supercoiling on transcription [1]. The inhibition of transcription at high negative supercoiling levels stems from the accumulation of hyper-negative supercoiled DNA behind the RNA polymerase, that hinders the progression of the transcription complex and thus may cause genome instability [14]. In this context, DNA topoisomerases of family IA perform an essential function in preventing the accumulation of hyper-negative supercoiled DNA, and their preferred substrate is often hyper-negatively supercoiled DNA. For example, *Drosophila melanogaster* topoisomerase IIIalpha relaxes hyper-negatively supercoiled DNA into supercoiled DNA and cannot relax supercoiled DNA to fully relaxed DNA [15]. Structural data have suggested that even if *Escherichia coli* DNA topoisomerase I (*Ec* TopoI) can relax negatively supercoiled DNA, its preference for hyper-negatively supercoiled DNA stems from the nature and the number of interactions between single-stranded DNA and its carboxy-terminal extremity that carries four cysteine zinc ribbon and zinc ribbon-like domains [16].

In the case of negatively supercoiled DNA, Wang suggested that tightly wound regions were prominent and that sharp bends exposing few bases and possibly becoming preferred substrates for nucleases could appear [1]. Thermodynamic considerations led him predict that, in contrast, hyper-negatively supercoiled DNA would expose denatured segments of significant length [1]. Over the past decades, experiments of electron microscopy, cryo-tomography and atomic force microscopy, various methods of simulation and 3D reconstruction, and biochemical assays have permitted to investigate the structural features (in terms of bend, kink, unwound regions, base flipping, asphericity, prolatness for example [17, 18]) of supercoiled DNA of various (i) sizes (ranging from mini-circles to plasmids [19]), (ii) sequences (*i*.*e*. cruciform [20], direct or inverted sequences [21,22], TATA sequence [23]), and (iii) supercoiling degrees (from positive to hyper-negative [24,25]) under various experimental conditions [18,26]. For instance, molecular dynamics simulations made it possible to appreciate the structural diversity adopted by a topoisomer of a given specific supercoiling level [25]. This method was however not able to precisely locate and identify the nucleotide(s) implicated in this structural diversity. As a consequence, at least two questions remained unanswered: (i) where on the sequence and at the nucleotide level, are these structural features located, and (ii), how these structural features influence the binding properties of proteins involved in DNA metabolism?

Our goal in the present study was therefore to investigate the specific structural changes induced by negative supercoiling and more specifically by hyper-negative supercoiling by using a very powerful method that permits a resolution at the nucleotide level. To this end, we performed two types of experiments: first, experiments that identify the nucleotide(s) that become(s) hyper-sensitive to or protected by enzymatic probes upon introduction of hyper-negative supercoils, and second, gel shift assays to evaluate the influence of hyper-negative supercoiling on the DNA binding properties of proteins. For both types of experiments, the DNA substrates used were double-stranded DNA mini-circles (dsMCs) for several reasons (reviewed in [27]). First, several methods (including our method [28]) of preparation of dsMCs make it possible to produce topoisomers whose topology ranges from relaxed to hyper-negatively or positively supercoiled forms of DNA [25,29–31]. Second, dsMCs have proved to be suitable substrates to characterize the biochemical activity of topoisomerases [30,32,33] and the structural properties of DNA, either naked [17,18,23,25,26,34] or assembled into chromatin [29,32,35–37]. Third, they have also been used as models of small topological domains such as DNA loops created by the packaging of the DNA or chromatin remodeling [38,39]. For the probing experiments, the probes used to estimate the proportion of unpaired bases and locate them were the methylating agent, dimethylsulfate (DMS), and two nucleases, the Nuclease SI and the DNAse I that detect and modify non-B DNA conformations and react with either single-stranded DNA (DMS and Nuclease SI), or double-stranded DNA (DNAse I). For the binding studies, the proteins able to bind single-stranded DNA were the human replication protein A (hRPA), *Escherichia coli* SSB (*Ec* SSB) and topoisomerase I from *Escherichia coli* (*Ec* TopoI). The biochemical assays were performed either in the absence or in the presence of divalent cations since specific (*e*.*g*. writhe) and non canonical structures (*e*.*g*. base flipping) may be favored under specific salt conditions [40]. Such cations indeed screen the negative charge of the DNA phosphates. As a result they (i) decrease DNA-DNA repulsion, (ii) induce a transition from a loosely to a tightly inter-wound conformation that correlates with a decrease of the effective DNA diameter and (iii) increase the writhe of the DNA [26,41].

Our results show an increased sensitivity of Nuclease SI and a decreased sensitivity of DNAse I with increasing negative supercoiling. At the nucleotide level, the structural changes responsible for this modulation of the nuclease activity are of low magnitude, located on both strands of the topoisomers and distributed along the entire sequence. No strong alteration of the structure (*e*.*g*. kink or sharp bending) can be detected. Our binding experiments performed in the absence of magnesium show that hRPA is sensitive to the negative supercoiling of the DNA and that the higher the negative supercoiling, the greater the number of proteins per dsMC. Since, in the absence of magnesium, the dsMCs topoisomers mainly differ by their twist, this result illustrates the twist-dependent binding activity of hRPA. In contrast, *Ec* TopoI exhibits a stronger binding on negatively and hyper-negatively supercoiled dsMCs than on relaxed DNA, but no significant difference in the binding activity among the supercoiled topoisomers can be measured. In a magnesium-containing buffer, topisomers of dsMCs can writhe and hRPA exhibits an unusual and more complex binding behavior, that does not show a direct correlation between the degree of negative supercoiling and the efficiency of binding. A similar behavior has been observed with *Ec* SSB.

## Materials and methods

### Materials

*Escherichia coli* topoisomerase I (*Ec* TopoI), T4 polynucleotide kinase (PNK), calf intestinal phosphatase, T4 DNA ligase, DNAse I, BamHI, BglII and HindIII were from New England Biolabs. [gamma -^32^P]-ATP was from Perkin Elmer. The human replication protein A (hRPA) was prepared in our laboratory [42,43]. Wheat germ Topoisomerase was from Inspiralis. Nuclease SI was from Promega. Proteinase K was from Sigma-Aldrich. *Escherichia coli* SSB (*Ec* SSB) was from USB.

### Preparation of ds DNA mini-circles (dsMCs) with a specific relative linking number

The procedure to prepare dsMCs of 235 base pairs with a specific linking number has been published elsewhere [28] and is presented on the S1 Fig. Briefly, the dephosphorylated DNA fragment of 235 base pairs was radiolabeled with T4 polynucleotide kinase [2 μM [gamma -^32^P]-ATP, 0.6 U microL^-1^ PNK, 30 min, 37°C in polynucleotide kinase buffer (70 mM Tris-HCl pH 7.6, 10 mM MgCl_2_, 5 mM DTT)] and ligated with T4 DNA ligase (2 U microL^-1^, 4 h, 16°C in T4 DNA ligase buffer (50 mM Tris-HCl pH 7.5, 10 mM MgCl_2_, 1 mM ATP, 10 mM DTT) in the presence of various concentrations of ethidium bromide (EtBr, from 0 to 20 microg mL^-1^). After ethidium bromide extraction, the covalently closed dsMCs were gel-purified on a 4% acrylamide (29:1 = acrylamide:bisacrylamide mass ratio) gel without chloroquine and recovered by electro-elution (S1 Fig). An analytical gel with chloroquine at 30 micro g mL^-1^ showing the sequential formation of the topoisomers as a function of [EtBr] present in the ligation step and their relative migration made it possible to attribute a specific relative linking number to each topoisomer based on the band counting method [44]. The relative linking number of 0 has been arbitrarily attributed to the dsMC obtained by ligation without ethidium bromide.

### Nuclease SI and DNAse I digestion of dsMCs

In the case of digestions of dsMCs with Nuclease SI, 0.5 nM of dsMC of indicated topology was incubated in 20 microL of Nuclease SI buffer (50 mM Tris-HCl pH 7.5, 50 mM NaCl, 20 mM MgCl_2_, 5% glycerol) for 5 min at 25°C before adding the enzyme at the indicated concentration. After 5 min incubation at 25°C, the reactions were stopped by adding EDTA (final concentration, 25 mM) and the samples were kept on ice. The digestions of dsMCs with DNAse I were performed in 20 microL of DNAse I buffer (40 mM Tris-HCl pH 7.9, 10 mM NaCl, 6 mM MgCl_2_, 1 mM CaCl_2_, 2 mM DTT). 0.5 nM of dsMC of indicated topology was then incubated for 5 min at 37°C in DNAse I buffer before adding the enzyme at the indicated concentration. After 15 min at 37°C, the reactions were stopped by adding EDTA (final concentration, 25 mM) and the samples were kept on ice. DNAse I and Nuclease SI were removed from the samples by adding SDS (final concentration, 0.5%), NaCl (final concentration, 0.5 M) and 1 volume of chloroform-isoamyl alcohol (24:1 v/v). The samples were vortexed at top speed for 2 min and centrifuged at 4°C for 10 min at 15,000 g. The aqueous upper phases were recovered and their DNA contents were precipitated with linear polyacrylamide carrier and 2.5 volumes of EtOH. The tubes were left on ice for at least 10 min and centrifuged for 20 min at 4°C at 15,000 g. After discarding the supernatant, the DNA pellets were rinsed with EtOH, dried before being analyzed by electrophoresis on a polyacrylamide gel.

### Treatment of dsMCs with DMS

Dilutions of DMS were freshly made in 100% EtOH. A 200 μL mixture of 0.5 nM dsMCs of indicated topology and 20 ng microL^-1^ of sonicated salmon sperm DNA was incubated with DMS at the indicated concentration in a DMS buffer (50 mM Na-cocadylate pH 8, 10 mM MgCl_2_, 1 mM EDTA) for 15 min at 20°C. The reaction was stopped by adding 50 microL of DMS stop buffer (50 mM Na-Acetate pH 7, 1 M beta -mercaptoethanol, 0.1 mg mL^-1^ tRNA). The sample was then precipitated with EtOH, treated with piperidine, lyophylized twice before being analyzed by electrophoresis on a polyacrylamide gel.

### Sequencing reactions

To sequence the single-stranded 235 nucleotide-long DNA, the 235 base pair-long fragment linearized by BamHI was completely digested by BglII or HindIII according to the manufacturer’s recommendations and sequenced according to the Maxam and Gilbert procedures specific for either guanines or guanines and adenines [45].

### Analysis of dsMCs in divalent cation-containing buffer

dsMCs of indicated topology (final concentration, 0.5 nM) were diluted in 20 mM Tris-0Ac pH 7.65 buffer supplemented with 50 mM NaOAc, 1 mM DTT and either Mg(OAc)_2_ (final concentration, 10 mM) or Ca(OAc)_2_ (final concentration, 10 mM) and incubated for 30 minutes at 37°C. At the end of the incubation, glycerol diluted in 50 mM Tris-HCl pH 7.5 was added to the samples at a final concentration of 5%. The samples were then analyzed by electrophoresis on a polyacrylamide gel.

### Interaction between dsMCs and hRPA or *Ec* SSB

The tests of interaction between dsMCs and hRPA were performed at 20°C for 10 min in 10 microL of hRPA binding buffer (20 mM Tris-HCl pH 7.5, 50 mM KCl, 1 mM DTT). When indicated, MgCl_2_ was added at a final concentration of 10 mM. Each dsMC of indicated topology was at 0.5 nM and the hRPA at the indicated concentration. When required, proteinase K was added, after 10 min interaction between dsMCs and hRPA, at a final concentration of 2 mg mL^-1^ for 10 min at 20°C. At the end of the interaction, glycerol was added at a final concentration of 5% and the samples were kept on ice before being analyzed by electrophoresis on a polyacrylamide gel. The interaction between dsMCs and *Ec* SSB has been tested under the conditions used with hRPA except for the binding buffers that had the following compositions. The magnesium-containing buffer contained 25 mM Tris-HCl pH 8, 50 mM NaCl, 10 mM MgCl_2_, 1 mM DTT. In the EDTA-containing buffer, MgCl_2_ was replaced by 1 mM EDTA. The T_-5_ topoisomer of dsMC was at 0.5 nM and the *Ec* SSB at the indicated concentration (0, 1, 3, 9 28, 83 ng microL^-1^).

### Interaction between dsMCs and *Ec* TopoI

The tests of interaction between dsMCs and *Ec* TopoI were performed at 37°C for 10 min in 10 microL of *Ec* TopoI binding buffer (20 mM Tris-HCl pH 7.5, 50 mM KCl, 25 mM EDTA, 1 mM DTT). Each dsMC of the indicated topology was at 0.5 nM and the *Ec* TopoI at the indicated concentration. At the end of the interaction, glycerol was added to the samples at a final concentration of 5% and samples were kept on ice before being analyzed by electrophoresis on a polyacrylamide gel.

### Conditions of gel analysis

All gels (except the sequencing gel) were made with an acrylamide:bisacrylamide mass ratio equal to 29:1. When the effect of calcium or magnesium on the electrophoretic migration of dsMC was investigated, the reaction products were loaded on a 4% polyacrylamide (14cmx14cmx1.5mm) gel made in 40 mM Tris-acetate pH 8.5, supplemented with either 10 mM Mg(OAc)_2_ or 10 mM Ca(OAc)_2_. Electrophoresis was performed at 4°C for 5 hours and at 120 V using a C.B.S. Scientific and Co system. The running buffer composed of 40 mM Tris-acetate pH 8.5, 10 mM Mg(OAc)_2_ or Ca(OAc)_2_ was continuously recycled during electrophoresis.

After the lyophylisation that follows the piperidine treatment, the pellets were redissolved in a denaturing loading buffer (TE (10 mM Tris-HCl pH 8, 1 mM EDTA) supplemented with 4% glycerol and 0.1M NaOH) and loaded onto a 4% polyacrylamide gel made in TBE 0.5x (44.5 mM Tris-Base, 44.5 mM boric acid, 1 mM EDTA) supplemented with 30 microg mL^-1^ chloroquine. Electrophoresis was performed at room temperature for 6 hours at 100 V.

When the extent of digestion of dsMCs by DNAse I or Nuclease SI as a function of enzyme concentration was investigated, after ethanol precipitation the pellets were redissolved in denaturing loading buffer (TE supplemented with 4% glycerol and 0.1M NaOH) and loaded onto a 6% polyacrylamide gel made in TBE 0.5x supplemented with 30 microg mL^-1^ chloroquine. Electrophoresis was performed at room temperature for 15 hours at 15 V.

When the profiles of digestion of dsMCs by DNAse I or Nuclease SI were investigated, after ethanol precipitation the pellets were redissolved in a formamide-containing loading buffer and loaded onto a 7%, 8% or 12% sequencing gel (acrylamide:bisacrylamide mass ratio equal to 19:1).

When the interaction between dsMCs and hRPA, *Ec* SSB or *Ec* TopoI was investigated, the samples were analyzed under native conditions on a 4% polyacrylamide gel made in TBE 0.5x (44.5 mM Tris-Base, 44.5 mM boric acid, 1 mM EDTA). When present, MgCl_2_ was added at a final concentration of 10 mM both in the gel and in the running buffer. Electrophoresis was performed at 4°C, for 4 hours and at 150 V.

### Quantification of the gels

When done, gels were quantified with the ImageQuant TL software.

## Results

Seven topoisomers of 235 base pairs with relative linking number (ΔLk) ranging from 0 to -6 (labeled T_i_ in what follows where i = relative linking number) were prepared to locate the nucleotides whose conformation changes in response to the introduction of negative supercoiling and to characterize the effect of negative supercoiling on the binding properties of hRPA and *Ec* TopoI, two essential proteins of the DNA metabolism.

### Activity of enzymatic and chemical probes on relaxed and hyper-negatively supercoiled dsMCs

**Fig 1:**
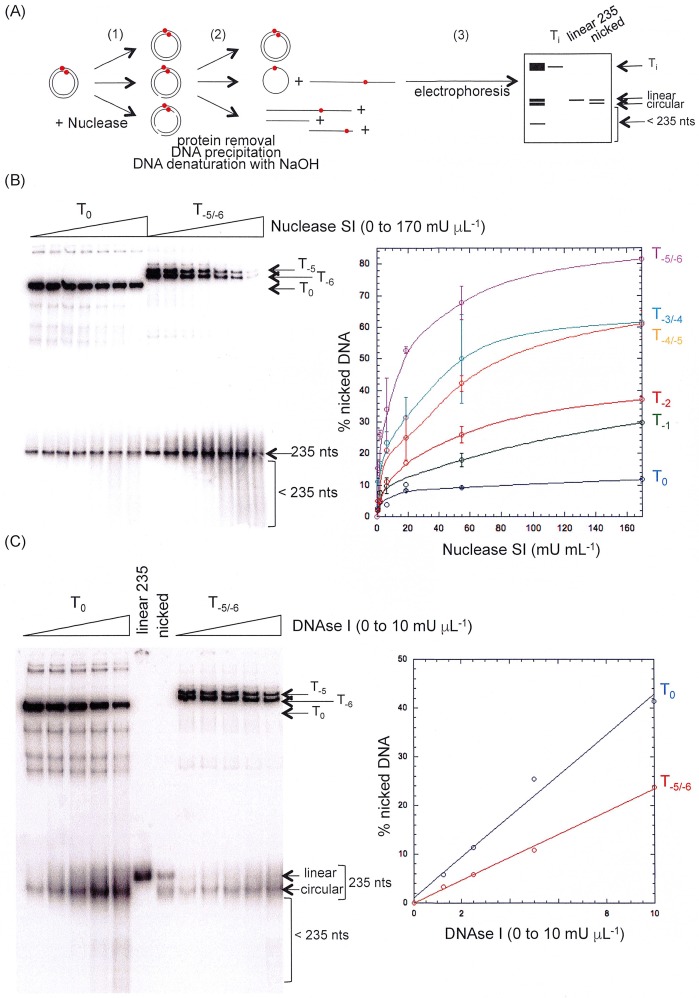
Digestion of topoisomers of dsMCs by nucleases. (A) Experimental scheme. The red-filled circle designates ^32^P. The different steps of the experiment are indicated: first (1), the digestion; second (2), the sample preparation for analysis by gel electrophoresis; third (3), electrophoresis. The topoisomers were incubated with increasing amounts of nuclease. At the end of the reaction, nucleases were removed from the reaction products. The DNAs were precipitated before being denatured by NaOH and resolved by electrophoresis on a polyacrylamide gel. Under these conditions, dsMCs were resolved from linear and circular single-stranded DNA. (B) Reactivity of topoisomers of dsMCs towards Nuclease SI. Left panel: picture of the gel showing the degradation of T_0_ and T_-5/-6_ topoisomers at increasing concentrations of Nuclease SI (0; 0.7; 2; 6.2; 18.5; 55; 170 mU microL^-1^). Right panel: Quantification of the degradation of the topoisomers at increasing concentrations of Nuclease SI. The % of nicked DNA is plotted as a function of Nuclease SI concentration. Error bars correspond to the standard errors calculated from three independent experiments. (C) Reactivity of topoisomers of dsMCs towards DNAse I. Left panel: picture of the gel showing the degradation of T_0_ and T_-5/-6_ topoisomers at increasing concentrations of DNAse I (0; 1; 2.5; 5; 10 mU microl^-1^). Right panel: Quantification of the degradation of the T_0_ and T_-5/-6_ topoisomers at increasing concentrations of DNAse I. The % of nicked DNA is plotted as a function of DNAse I concentration. A duplicate of this experiment has been done and gave similar results in terms of the difference of reactivity of the DNAseI between the T_0_ and T_-5/-6_ topoisomers. “nts” signifies nucleotides.

Conformational changes of the dsMCs associated with the introduction of various levels of writhe and compaction might be detected by enzymatic and chemical probes. Nuclease SI is specific of single-stranded DNA, whereas DNAse I is specific of double-stranded DNA and both nucleases can detect non canonical structures into double-stranded DNA [12–14,16–18,46–48]. To locate the nucleotides whose conformation changed in response to the introduction of negative and hyper-negative supercoiling, we first checked the sensitivity of the radiolabeled topoisomers of dsMCs with these two protein probes, Nuclease SI and DNAse I. It should be noted that the Nuclease SI digestion was performed at neutral pH by adjusted experimental conditions as recommended [49]. Each topoisomer was incubated with increasing amounts of nuclease for a given period of time and at a given temperature (Fig 1A for an experimental scheme). Nucleases were removed from the reaction products and DNA was precipitated before being denatured by NaOH and analyzed by electrophoresis on a polyacrylamide gel. Under these conditions, dsMCs were resolved from single-stranded DNAs, either circular or linear. The results show a decrease of the amount of substrate and an accumulation of ≤ 235 nucleotide-long DNA fragments as a function of Nuclease SI concentration (Fig 1B, left panel). Quantification of the gel indicate that the higher the negative supercoiling, the more active the Nuclease SI (Fig 1B, right panel). In contrast, the results obtained with DNAse I show that two times more enzyme is required to nick a mixture of topoisomers T_-5_ and T_-6_ as efficiently as the topoisomer T_0_ (Fig 1C). For both experiments, the reaction products consist of fragments whose length is ≤ 235 nucleotides, revealing multiple cuts made by the nucleases per dsMC. Similar results were obtained by others [19].

**Fig 2:**
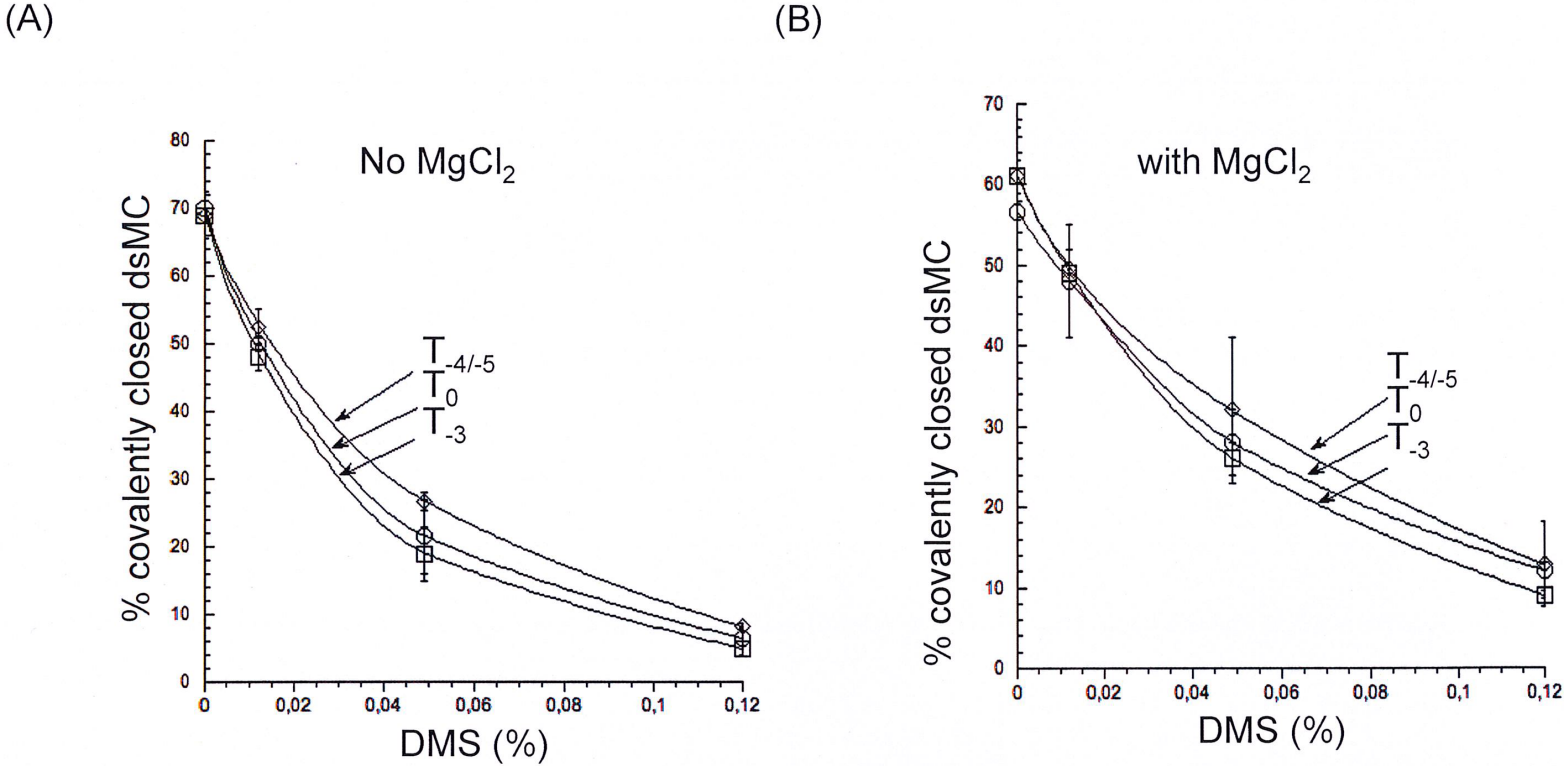
Reactivity of the DMS on the topoisomers of dsMCs in the absence or in the presence of magnesium. (A) Comparison of the reactivity of three topoisomers (T_0_, T_-3_, T_-4/-5_) with DMS in the absence of magnesium. (B) Comparison of the reactivity of three topoisomers (T_0_, T_-3_, T_-4/-5_) with DMS in the presence of magnesium. [DMS] ranges from 0 to 0.125%. The % of covalently closed dsMCs is represented as a function of [DMS]. Error bars correspond to the standard errors calculated from three independent experiments.

In contrast to the results obtained with the enzymatic probes, the different topoisomers exhibited the same reactivity towards DMS, independently of the presence or not of divalent cations (Fig 2) revealing a difference of sensitivity between chemical and enzymatic probes.

Altogether, these results indicate that hyper-negatively supercoiled dsMCs are more accessible to the Nuclease SI digestion than the relaxed topoisomer, possibly exposing additional and/or better substrates. In contrast, relaxed dsMCs are slightly better substrates for DNAse I than hyper-negatively supercoiled dsMCs, in agreement with the Nuclease SI results, given the opposite specificities of the two nucleases.

### Location of the nucleotides sensitive to hyper-negative supercoiling

**Fig 3:**
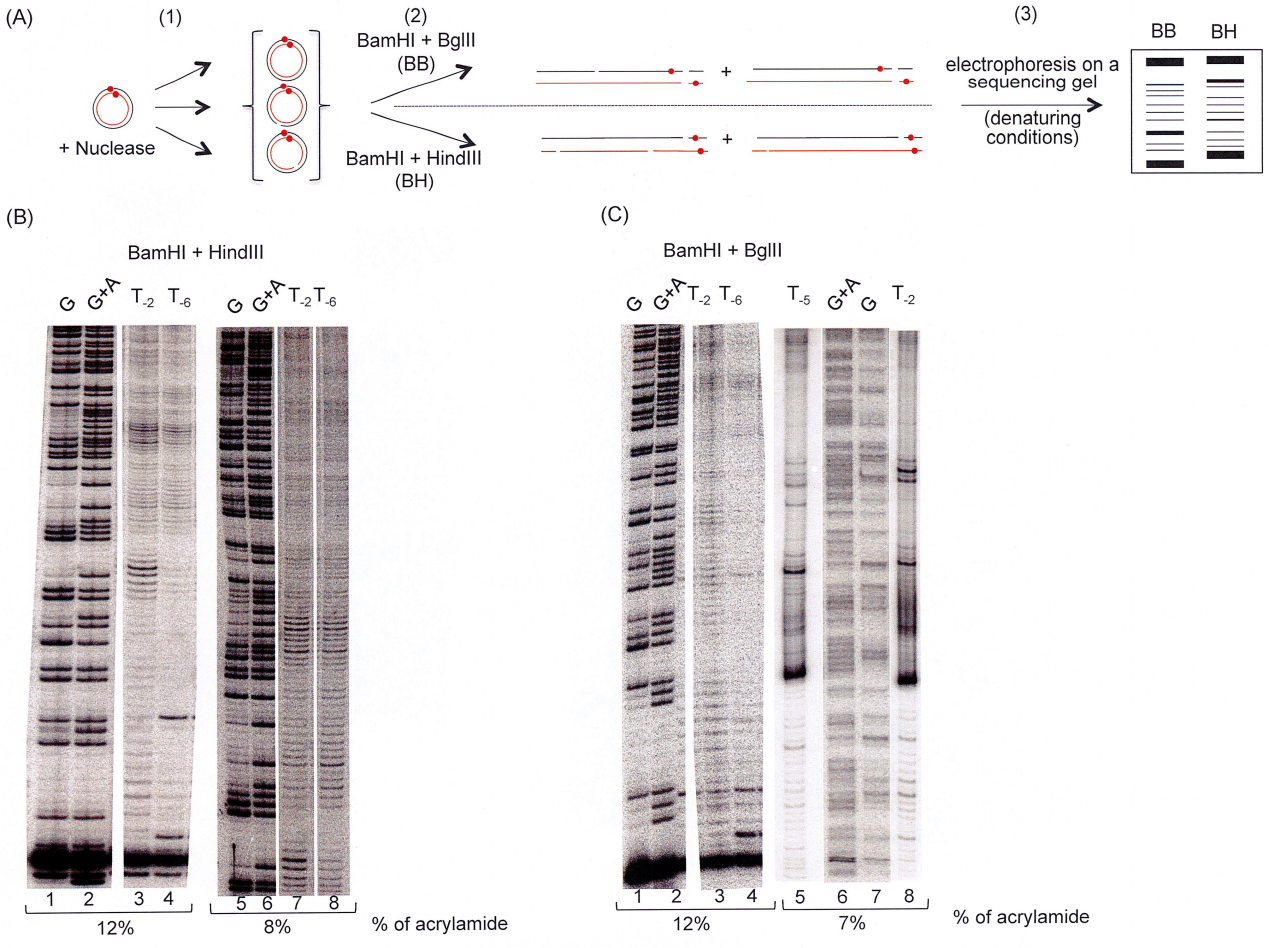
Sites of structural changes induced by the hyper-negative supercoiling detected by Nuclease SI. (A) Experimental scheme. The red-filled circle designates ^32^P. The different steps of the experiment are indicated: first (1), the digestion by the Nuclease SI; second (2), the digestion by (BamHI + BglII) or (BahmHI + HindIII); third (3), electrophoresis on a sequencing gel. (B) The enzymatic probe used to map the fine structure of the T_-2_ and T_-6_ topoisomers is Nuclease SI. Nuclease SI is at 2 mU microL^-1^ and DNA at 0.5 nM. After the Nuclease SI reaction, the samples are treated to remove the proteins. The DNAs are precipitated and submitted to the BamHI+HindIII double digestion to only visualize DNA fragments from one of the two radiolabeled strands. The reaction products are analyzed on two different sequencing gels (8% to see long DNA fragments, 12% to see short DNA fragments) as indicated. G and G+A lanes correspond to the products of the Maxam and Gilbert reactions to identify specifically the guanines (G lanes; lanes 1 and 5) or the guanines and adenines (G+A lanes; lanes 2 and 6). (C) Same as 3B except that the samples are submitted to the BglII+BamHI double digestion to only visualize DNA fragments from the complementary radiolabeled strands. The reaction products are analyzed on two different sequencing gels (7% to see long DNA fragments, 12% to see short DNA fragments) as indicated. G and G+A lanes correspond to the products of the Maxam and Gilbert reactions to identify specifically the guanines (G lanes; lanes 1 and 7) or the guanines and adenines (G+A lanes; lanes 2 and 6).

The difference of reactivity of the topoisomers with Nuclease SI and DNAse I having been established and shown to be significantly different, especially in the case of the Nuclease SI, we next investigated which nucleotide(s) on the dsMCs sequence became hyper-sensitive or hypo-sensitive to Nuclease SI upon introduction of hyper-negative supercoiling in the dsMCs. To this end, the concentration of nuclease that permitted to digest less than 15% of the initial substrate (corresponding to on average of one nick per DNA molecule, at most) was determined, and this nuclease concentration was used to compare the profiles of digestion of two topoisomers of dsMCs, T_-2_ and T_-5/-6_ for the Nuclease SI digestion (Fig 3A for an experimental scheme). After the nuclease digestions, the reaction products were digested by either the BamHI/BglII or the BamHI/HindIII restriction enzyme couple (Fig 3A) in order to analyze only one of the two radiolabeled strands of the dsMCs on a sequencing gel (our procedure to prepare dsMCs indeed leads to dsMCs radiolabeled on both strands (see Materials and Methods and S1 and S2 Figs)).

Results show that the digestion profiles of T_-2_ and T_-5/-6_ by the Nuclease SI are overall quite similar. Phosphodiester bonds becoming slightly hyper-sensitive upon supercoiling change can be located on either topoisomer and on either of the two strands of each dsMCs (Fig 3B). Similar digestion profiles of T_0_ and T_-5/-6_ topoisomers with the DNAse I were obtained (S3 Fig). The small difference of reactivity of the phosphodiester bonds as a function of negative supercoiling suggests very subtle conformational changes between T_i (i = 0 or -2)_ and T_-5/-6_ topoisomers.

### Partitioning between twist and writhe in the topoisomers

**Fig 4:**
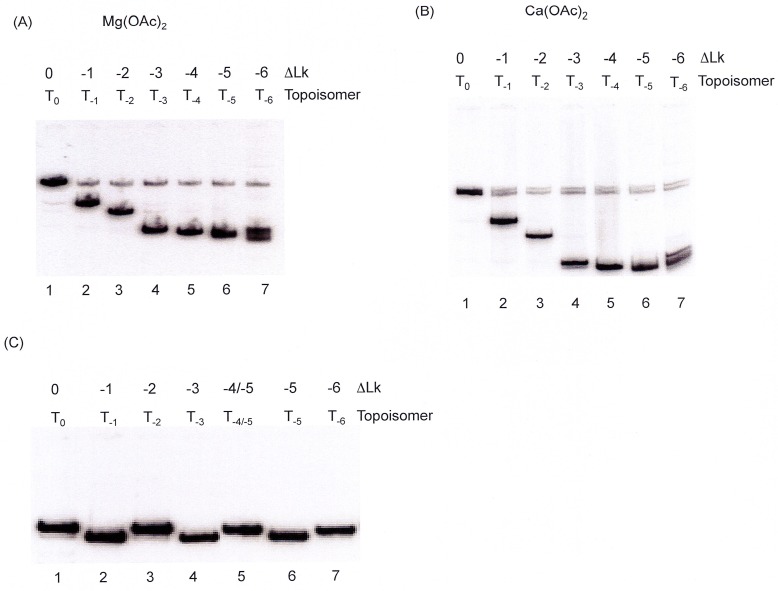
Effect of magnesium or calcium on the conformations of the topoisomers of dsMCs. (A) Electrophoretic migration profile of the seven topoisomers in the presence of magnesium. Magnesium (10 mM) is present in the native polyacrylamide gel and in the running buffer. (B) Electrophoretic migration profile of the seven topoisomers in the presence of calcium. Calcium (10 mM) is present in the native polyacrylamide gel and in the running buffer. (C) Electrophoretic migration profile of the seven topoisomers on a native gel made in TBE 0.5x. The topoisomers differ by their relative linking number. In all figures, ΔLk ranges from 0 (T_0_ topoisomer, lane 1) to -6 (T_-6_ topoisomer, lane 7). Electrophoreses are performed at 4°C with buffer being recycled.

Negative supercoiling can be absorbed by the twist and/or the writhe. Gel electrophoresis under native conditions can resolve topoisomers that have different extents of writhe. This method made it possible to establish that negative supercoiling in the absence of divalent cations, partitioned almost exclusively into untwisting of the DNA helix and that the presence of salt (especially divalent cations) modified the relative proportion of twist, writhe and compaction in the DNA [26,31,40,50]. The Nuclease SI and DNAse I reactions being performed in the presence of divalent cations, we characterized the relative mobilities of our seven topoisomers by gel electrophoresis in the presence of magnesium or calcium (Fig 4A and 4B).

Results of the electrophoresis indicate that the T_0_ topoisomer has the slowest mobility, the T_-3_, T_-4_, T_-5_ and T_-6_ the highest mobility. The mobility of the T_-1_ and T_-2_ dsMCs is intermediate. This data suggests the existence of at least two writhe sites and a high degree of compaction for hyper-negatively supercoiled dsMCs in the presence of divalent cations. In contrast, in the absence of divalent cations, the similar mobilities of our topoisomers suggest that they mostly differ by their twist (Fig 4C), as expected.

### Interaction between topoisomers of dsMCs and single-stranded DNA binding proteins in the absence of divalent cations

**Fig 5:**
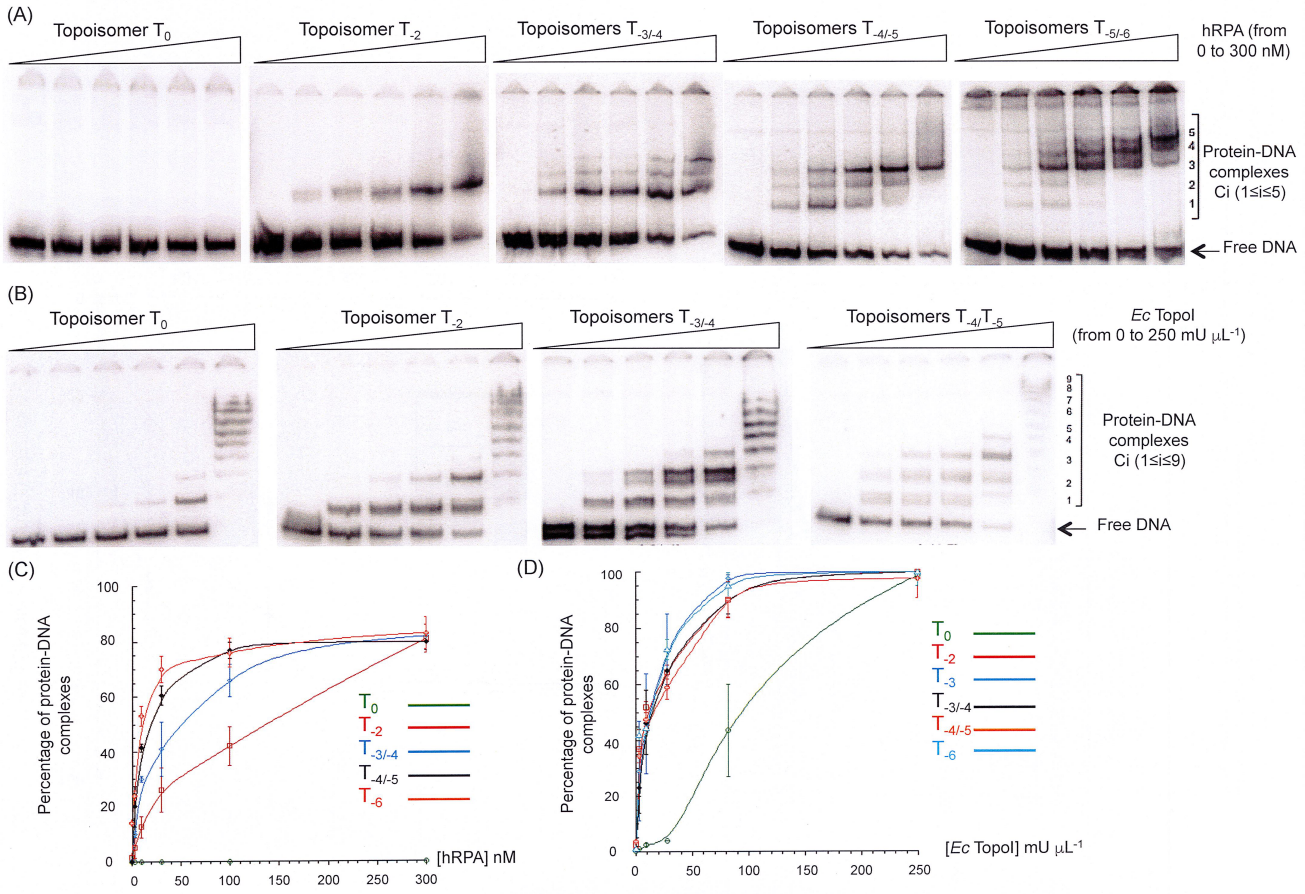
Binding of hRPA and *Ec* TopoI to the topoisomers of dsMCs in the absence of magnesium. Each dsMC of indicated topology is at 0.5 nM and the DNA binding protein at the indicated concentration. For both titrations, the samples are analyzed by electrophoresis on native 4% polyacrylamide gels (29:1 = acrylamide:bisacrylamide mass ratio) made in TBE 0.5x. Electrophoresis is performed at 150 V, 4°C and for 4 h. Quantification of the gels is made using the ImageQuant TL software. (A) Binding of hRPA. [hRPA] ranges from 0 to 300 nM (0; 3; 10; 30; 100; 300 nM). (B) Binding of *Ec* TopoI. [*Ec* TopoI] ranges from 0 to 250 mU microL^-1^ (0; 3; 9; 28; 81; 250 mU microL^-1^). (C) Quantification of Fig 5A. The percentage of hRPA-DNA complex is shown as a function of hRPA concentration. (D) Quantification of Fig 5B. The percentage of *Ec* TopoI-DNA complex is shown as a function of *Ec* TopoI concentration.

The probing experiments performed with Nuclease SI and DNAse I identified subtle conformation changes related to changes in DNA supercoiling that spread along the entire sequence (Fig 3). The gel electrophoresis experiments pinpointed various degrees of compaction and twist in the family of topoisomers (Fig 4). We next wondered whether a protein that binds single-stranded DNA could detect the structural differences among the topoisomers of dsMCs and more specifically in the extent of locally unwound regions. In the past, various biochemical bulk assays (see for example [51–53]), electron microscopy [13], AFM [54] and single-molecule [55] experiments used various single-stranded DNA binding proteins as a reporter, stabilizer or protector of single-stranded DNA. Consequently, the interaction between each topoisomer T_i_ and two proteins known to bind single-stranded DNA, the hRPA and *Ec* TopoI, was characterized. Since the binding studies were performed in the absence of divalent cations, relaxation of the negatively supercoiled dsMCs by the *Ec* TopoI was prevented. The topoisomers T_0_, T_-2_, mixtures of topoisomers T_-3_/T_-4_, T_-4_/T_-5_ and T_-5_/T_-6_ were incubated with increasing amounts of hRPA or *Ec* TopoI, and the resulting products were resolved on a polyacrylamide gel under native conditions (Fig 5A and 5B).

The results indicate that with concentrations of hRPA ranging from 0 to 300 nM, up to five different protein-DNA complexes (called C1 to C5) are resolved on a polyacrylamide gel (Fig 5A). It is possible that these complexes differ by the number of hRPA molecules bound per DNA molecule, the C1 complex migrating the fastest containing less hRPA molecules per dsMC than the C5 complex migrating the slowest. No protein-DNA complex is formed with the topoisomer T_0_, and a single species (C1) is assembled with the topoisomer T_-2_ (Fig 5A). The C2 and C3 complexes start forming with the mixture of topoisomers T_-3_/T_-4_ and the C3 complex is the most abundant complex formed with the mixture of topoisomers T_-4_/T_-5_ at the highest hRPA concentration (300 nM) (Fig 5A). The C4 and C5 complexes only form with the mixture of topoisomers T_-5_/T_-6_. Similar results are obtained with *Ec* TopoI, except that (i) within the range of concentrations used, up to 9 protein-DNA complexes are resolved on a polyacrylamide gel, (ii) *Ec* TopoI can bind to the topoisomer T_0_, and (iii) the largest complexes (Ci, 6≤ i ≤ 9) start forming with the topoisomer T_0_ (Fig 5B). Quantification of the gels indicates that the more negatively supercoiled the dsMCs, the better the binding of hRPA and that the best binding is obtained with T_-6_ topoisomers (Fig 5C). *Ec* TopoI binds equally well negatively and hyper-negatively supercoiled dsMCs but better than relaxed dsMCs (Fig 5D). These results illustrate different binding modes of hRPA and *Ec* TopoI. Altogether, our results show that without magnesium hRPA and *Ec* TopoI are sensitive to the negative supercoiling of the DNA and that the higher the negative supercoiling, the greater the number of proteins per dsMC. As in the absence of magnesium, the topoisomers mostly differ by their twist, our results show therefore that binding of hRPA and *Ec*TopoI is twist-dependent.

### Interaction between topoisomers of dsMCs and hRPA in the presence of divalent cations

**Fig 6:**
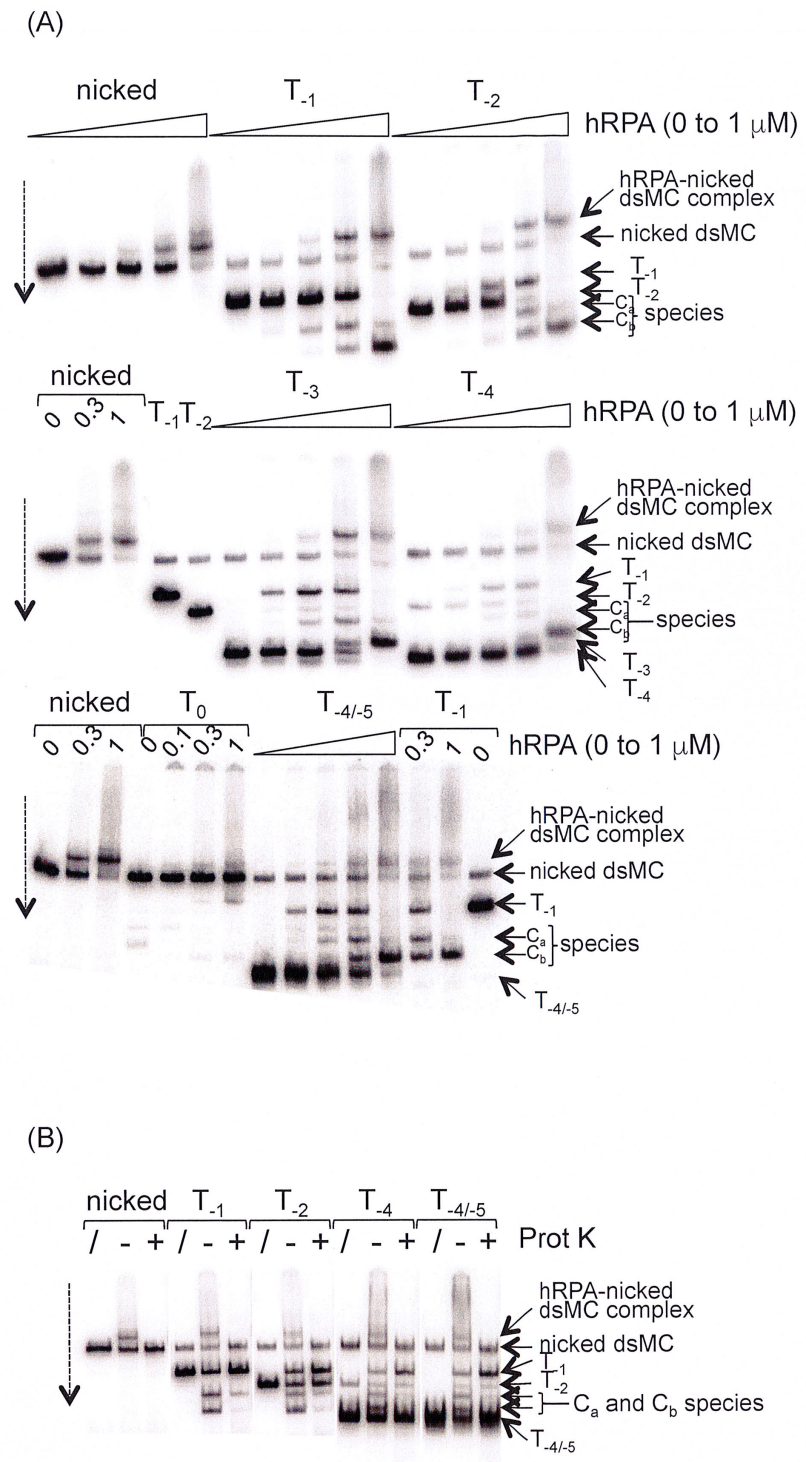
Interaction between hRPA and topoisomers of dsMCs in the presence of magnesium. The dashed arrows indicate the direction of the electrophoretic migration. (A) The topoisomers of indicated topology are incubated with increasing amounts of hRPA. [hRPA] ranges from 0 to 1 microM (final concentration, 0; 30; 100; 300; 1000 nM). The reaction products are analyzed by electrophoresis on a native 4% polyacrylamide (29:1 = acrylamide:bisacrylamide mass ratio) gel made in TBMg (44.5 mM Tris-Base, 44.5 mM boric acid, 10 mM MgCl_2_). Electrophoresis is performed at 150 V, 4°C and for 4 hours. Three complexes form upon mixing a given topoisomer with hRPA: the hRPA-nicked dsMC complex, the C_a_ and the C_b_ complexes. (B) Same as (A) with a final concentration of hRPA of 300 nM. Samples were (“+” lanes) or not (“-”lanes) treated with proteinase K (Prot K) before their analysis by electrophoresis on a native polyacrylamide gel. The three protein-DNA complexes that assemble on the nicked, T_-1_, T_-2_, T_-4_ and T_-4/-5_ DNA are sensitive to Proteinase K.

Since in the absence of magnesium, the extent of binding of hRPA depended on supercoiling (Fig 5A and 5C), we investigated whether this dependency persisted in a magnesium-containing buffer. To this end, nicked, T_0_, T_-1_, T_-2_, T_-3_, T_-4_ and a mixture of T_-4_ and T_-5_ DNA were incubated with increasing amounts of hRPA and the products were resolved by electrophoresis on a polyacrylamide gel (Fig 6A).

In these experiments, magnesium was not only present in the reaction buffer but also in the polyacrylamide gels and in the electrophoresis buffer. The results (Fig 6A) indicate that hRPA interacts with nicked dsMC, giving rise to a complex (hRPA-nicked dsMC complex) that migrates more slowly than naked DNA. With the T_-1_ topoisomer, two radiolabeled species (C_a_ and C_b_, Fig 6A) that migrate faster than naked dsMC can be isolated. The hRPA-nicked dsMC complex is also formed due to the presence of nicked dsMCs in our preparation. The same species are also formed with the other topoisomers of dsMCs. We note the appearance of T_-1_ topoisomers in the hRPA titration performed with T_i_ (i ≤ -2) topoisomers that is probably due to a small contamination of our preparation of hRPA by *Escherichia coli* topoisomerases.

To characterize the C_a_ and C_b_ species, we first checked if they were sensitive to proteinase K treatment. The radioactive bands corresponding to C_a_ and C_b_ complexes and the hRPA-nicked dsMC complex disappear almost completely upon treatment with the proteinase K (Fig 6B), indicating that the C_a_ and C_b_ species are nucleoprotein complexes. We next quantified the interaction at 300 nM hRPA. No clear correlation between the proportion of hRPA-DNA complexes and the degree of negative supercoiling (Table 1) could be observed: the proportion of the C_a_ and C_b_ species varies between 19% with the T_-4_ topoisomer and 44% with the T_-2_ dsMC (Table 1, squares colored in grey).

**Table 1:** Percentage of the different species at equilibrium of reaction between hRPA at 300 nM and the topoisomer T_i_.

	hRPA + T_-1_		hRPA + T_-2_		hRPA + T_-4_		hRPA + T_-4/-5_	
T_-1_	65 +/- 4		36 +/- 7		12.5 +/- 7		21.5 +/- 3	
T_-2_			20 +/- 13		1.5 +/- 0.7		2 +/- 0.5	
C_a_	18 +/- 1.5	C_a_ + C_b_ = 35	16 +/- 4	C_a_ + C_b_ = 44	7.5 +/- 2	C_a_ + C_b_ = 19	11 +/- 2	C_a_ + C_b_ = 34
C_b_	17 +/- 3		28 +/- 4		11.5 +/- 5		23 +/- 6	
T_-4_					62 +/- 4			
T_-4/-5_							43 +/- 2	

The reaction contains 10 mM Magnesium. Calculated values come from two to five independent experiments. The calculated error corresponds to the standard error. The percentage of C_a_ + C_b_ complexes is highlighted in the boxes colored in grey.

**Fig 7:**
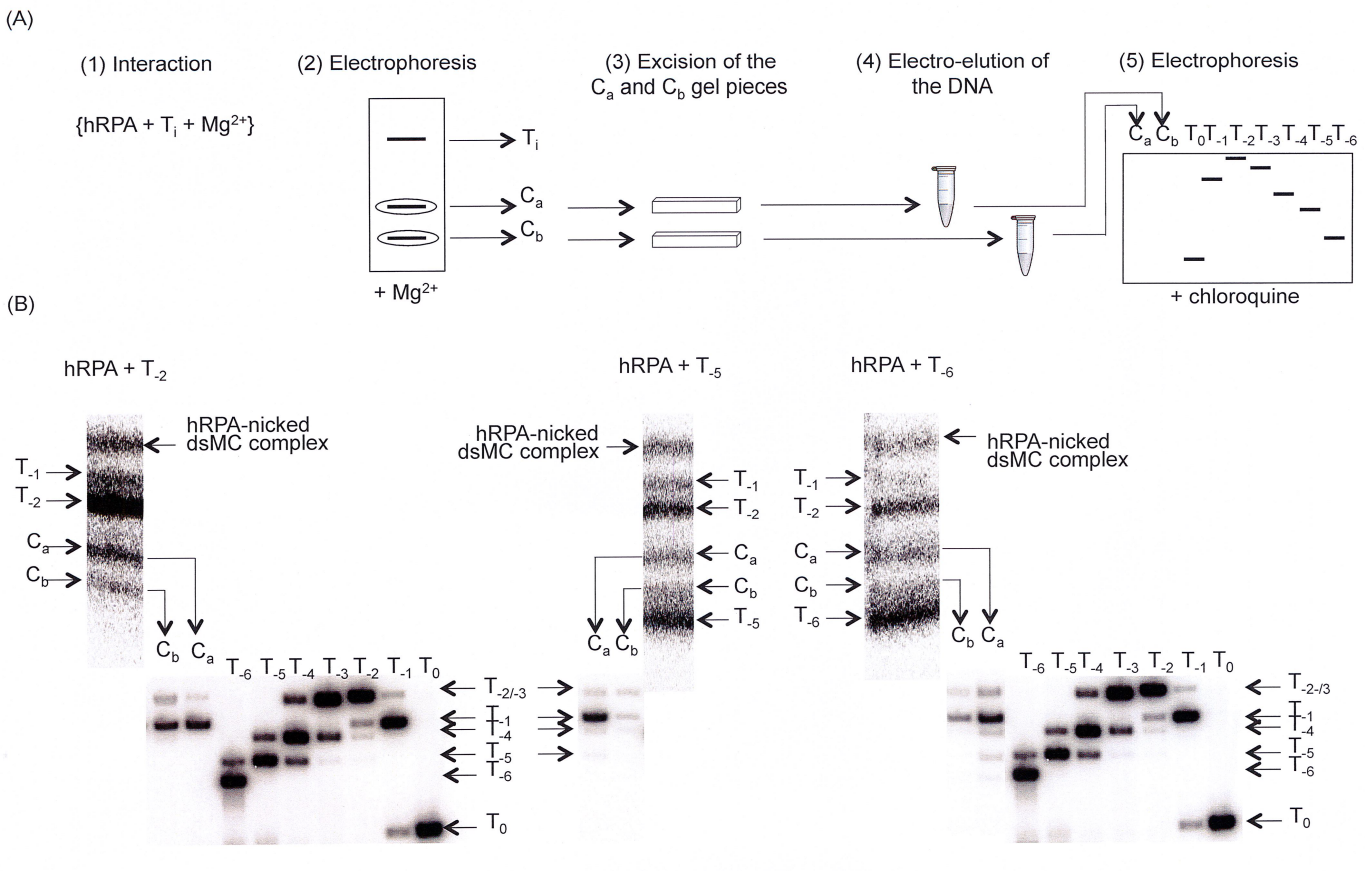
Topology of the DNA in the hRPA-dsMC complexes formed in the presence of magnesium. (A) Experimental scheme. The experiment is a succession of 5 steps: interaction between dsMC and hRPA in the presence of Mg^2+^ (1), electrophoresis under native conditions to separate nucleoprotein complexes from naked dsMCs (2), excision of C_a_ and C_b_ gel pieces after exposure of the gel under a ^32^P sensitive screen (3), electro-elution (4) and electrophoresis under conditions that resolve topoisomers (5). (B) Topology of the DNA in the C_a_ and C_b_ complexes. *Upper part of the figure*: hRPA (final concentration, 300 nM) is incubated with the T_-2_ (left panel), T_-5_ (middle panel) or T_-6_ (right panel) topoisomer (final concentration, 0.5 nM) and the different molecular species are resolved by electrophoresis on a native 4% polyacrylamide (29:1 = acrylamide:bisacrylamide mass ratio) gel made in TBMg (44.5 mM Tris-Base, 44.5 mM boric acid, 10 mM MgCl_2_). Electrophoresis is performed at 150 V, 4°C and for 4 h. *Lower part of the figure*: The DNAs contained in the gel bands corresponding to the C_a_ and C_b_ complexes are electro-eluted and analyzed by electrophoresis on a chloroquine-containing gel (6% acrylamide; 29:1 = acrylamide:bisacrylamide mass ratio; 30 microg mL^-1^ of chloroquine; TBE 0.5x). The dsMC interacting with hRPA is either T_-2_ (left part of the figure), T_-5_ (middle part of the figure) or T_-6_ (right part of the figure).

The topological state of the DNA in the C_a_ and C_b_ complexes formed with T_-2_, T_-5_ and T_-6_ topoisomers was next examined (Fig 7).

For this, the T_-2_, T_-5_ and T_-6_ topoisomers were incubated with hRPA in a magnesium-containing buffer (step 1. on Fig 7A) and species were resolved on polyacrylamide gel (step 2. on Fig 7A). After exposure of the gel on a ^32^P-sensitive screen, the gel bands corresponding to the C_a_ and C_b_ complexes were excised (step 3. on Fig 7A), their DNA content was electro-eluted (step 4. on Fig 7A) and their topological state was analyzed by electrophoresis on a chloroquine-containing gel able to resolve the topoisomers (step 5. on Fig 7A). The results show that the C_a_ and C_b_ complexes are clearly enriched in the T_-1_ dsMC (Fig 7B). We measured the proportion of each unbound dsMC in the reaction mixture and the proportion of each topoisomer in the C_a_ and C_b_ complexes (Table 2). No direct correlation between the proportion of unbound dsMCs in the reaction mixture and the proportion of topoisomers in the C_a_ and C_b_ complexes could be established.

**Table 2:** Relative percentage of unbound dsMCs at the equilibrium reaction and relative percentage of dsMCs associated with hRPA in the C_a_ and C_b_ complexes.

	hRPA + T_-2_			hRPA + T_-5_			hRPA + T_-6_		
	At equilibrium of the reaction	In C_a_	In C_b_	At equilibrium of the reaction	In C_a_	In C_b_	At equilibrium of the reaction	In C_a_	In C_b_
T_-1_	7	97	86	9.5	90	42	4	84	78
T_-2_	93	3	14	40			36		
T_-2/-3_					4.5	51		14	11
T_-4_					5	7		2	7
T_-5_				50.5	0.5				3
T_-6_							60		1

The reaction contains 10 mM Magnesium.

**Fig 8:**
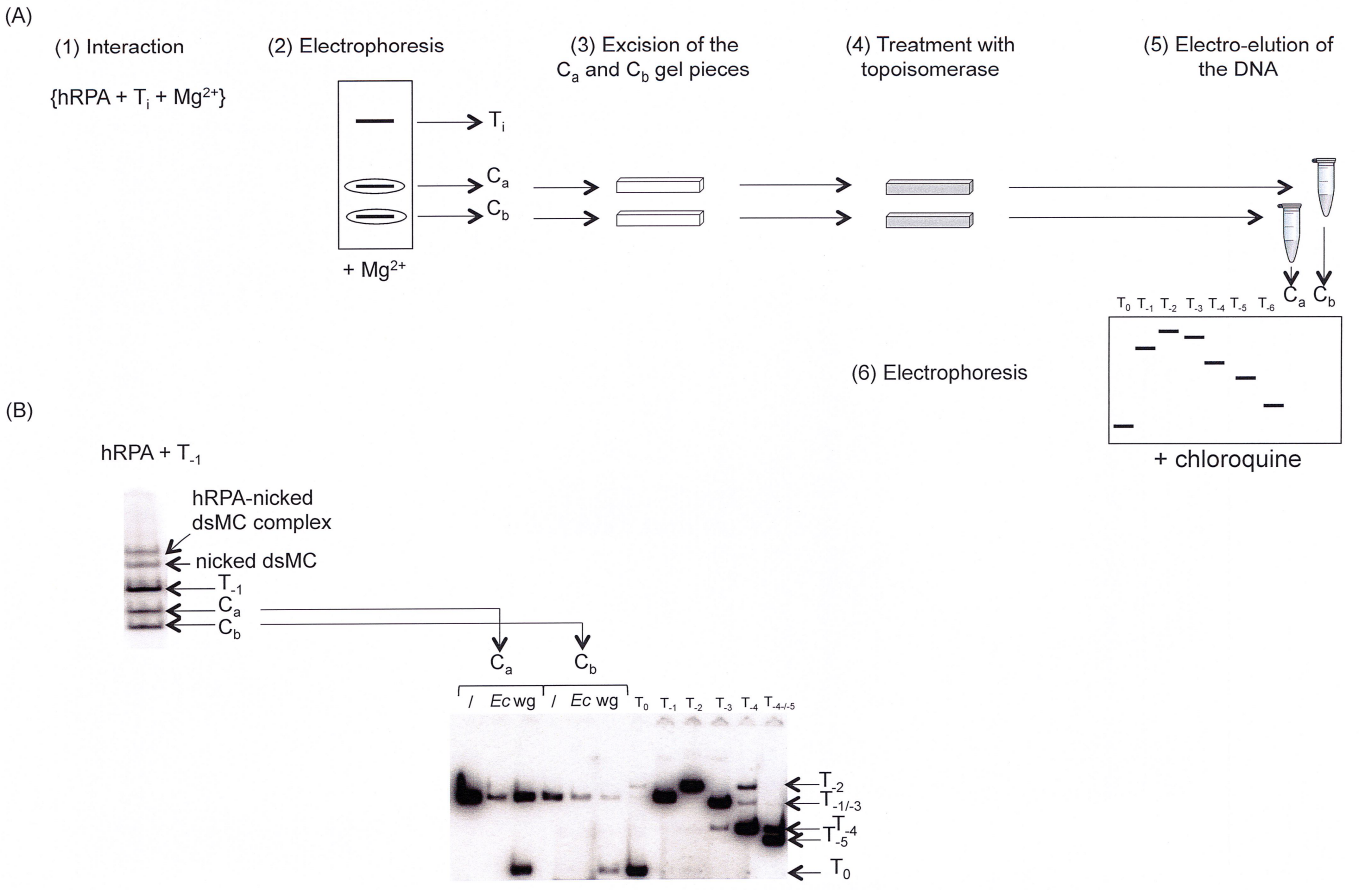
Topology of the hRPA-dsMC complexes formed in the presence of magnesium probed by the *Ec* TopoI and the wheat germ Toposiomerase. (A) Experimental scheme. The experiment is a succession of 6 steps: interaction between T_-1_ dsMC and hRPA in the presence of Mg^2+^ (1), electrophoresis under native conditions to separate nucleoprotein complexes from naked topoisomers (2), excision of C_a_ and C_b_ gel pieces after exposure of the gel under a ^32^P sensitive screen (3), “in gel” topoisomerase assay (4), DNA electro-elution (5) and electrophoresis under conditions that resolve topoisomers (6). (B) Determination of the topological state of the dsMC in the C_a_ and C_b_ complexes assembled on the T_-1_ topoisomer. *Upper part of the figure*: The species formed upon incubating hRPA (300 nM) with the T_-1_ topoisomer (0.5 nM) are resolved by electrophoresis on a 4% native polyacrylamide (29:1 = acrylamide:bisacrylamide mass ratio) gel made in TBMg (44.5 mM Tris-Base, 44.5 mM boric acid, 10 mM MgCl_2_). *Lower part of the figure*: The gel pieces corresponding to the C_a_ and C_b_ complexes are treated with *Ec* TopoI (*Ec*) or wheat germ Topoisomerase (wg) before performing an electro-elution to recover the DNA. The DNA recovered is precipitated and analyzed by electrophoresis on a chloroquine-containing gel (6% acrylamide; 29:1 = acrylamide:bisacrylamide mass ratio; 30 microg mL^-1^ of chloroquine; TBE 0.5x).

Finally, to investigate whether the topology of the dsMCs changed upon binding of hRPA, an “in gel” relaxation assay was carried out. We incubated the T_-1_ topoisomer with hRPA in a magnesium-containing buffer (step 1. on Fig 8A), separated the interaction species by gel electrophoresis (step 2. on Fig 8A), cut the gel bands corresponding to the C_a_ and C_b_ species (step 3. on Fig 8A), incubated them with either the *Ec* TopoI or the wheat germ Topoisomerase (step 4. on Fig 8A), electro-eluted the dsMCs from the gel pieces (step 5. on Fig 8A), and finally measured the relative linking numbers of the topoisomerase products by electrophoresis on a polyacrylamide gel containing chloroquine (step 6. on Fig 8A).

The results show that the products of the “in gel” relaxation reaction are T_-1_ in case of the treatment of the complex C_a_ or C_b_ with the *Ec* TopoI and T_0/-1_ in case of the treatment of the complex C_a_ or C_b_ with the wheat germ Topoisomerase (Fig 8B). Knowing that *Ec* TopoI has no activity on T_-1_ topoisomers and that the wheat germ Topoisomerase relaxes T_-1_ topoisomers into T_0_ topoisomers [28], our results indicate that the binding of hRPA on T_-1_ topoisomer does not induce a topological change in the dsMC.

## Discussion

Our goals in this study were first to locate at the nucleotide level, any structural changes induced by the hyper-negative supercoiling, by using two enzymatic probes, Nuclease SI and DNAse I, and second to characterize the effect of hyper-negative supercoiling on the binding of two proteins known to bind single-stranded DNA, the hRPA heterotrimer and *Ec* TopoI. These two effects of hyper-negative supercoiling were investigated in the absence and in the presence of magnesium since divalent cations influence the structure of DNA, and more specifically its topology. Due to its base pair resolution, our study brings an additional stone to the field of topology by complementing other methods including molecular dynamics simulations and 3D reconstructions that provide a global picture of the structural deformation induced by DNA supercoiling without giving access to the identity of the nucleotide(s) that become mis-paired or unpaired upon DNA supercoiling. Furthermore by comparing DNA binding properties of proteins in the absence or in the presence of magnesium, our results illustrate the influence of magnesium on the DNA binding behavior of single-stranded DNA binding proteins.

The DNA substrates that we used to conduct this study were topoisomers of dsMCs of 235 base pairs and spanned differences of topology ranging from relaxed (T_0_) to hyper-negatively supercoiled DNA (T_-3_, T_-4_, T_-5_ and T_-6_). Our results first, show that the negative supercoiling strongly stimulates the nuclease SI activity (Fig 1) and second, suggest differences of compaction and writhe among our topoisomers in a magnesium- or calcium- containing environment (Fig 4). When analyzed at the nucleotide level, our analyses indicate that the structural changes revealed by the difference of reactivity of the Nuclease SI on the phosphodiester bonds of the dsMCs are of low magnitude, located on the two strands of the topoisomers, and with single nucleotides or few nucleotide regions becoming either slightly hyper- or slightly hypo-sensitive upon introduction of negative supercoiling (Fig 3). All together our results suggest that the negative supercoiling induces subtle conformational changes that distribute along the entire sequence of the dsMCs without any strong sequence preference. The effects of these subtle conformation changes sum up to lead to the strong stimulation of the Nuclease SI activity by negative supercoiling. The results obtained with both types of probes are consistent with the identification of a limited number (≤ 2) of regions of ≈ 500 base pairs cleaved by Nuclease SI on the 5224 bp-long SV40 DNA [56]. It should however be noted that the use of radiolabeled dsMCs that carry appropriate restriction sites gives a much more detailed picture of the DNA conformation of topoisomers, with a base pair resolution. Similar studies performed in the *absence* of divalent cations used other DNA substrates, probes and methods to quantify the non-base-paired regions in supercoiled DNAs [56–59]. They suggested the presence of localized regions of denaturation, that in the case of the supercoiled DNA of the bacteriophage PM2 overlapped with the earliest melting regions of the relaxed DNA [59]. Similarly, in the case of the dsMCs produced by the ligation of a 75 base pair fragment induced by the HMGB2 (high mobility group of family B2), a denatured region of ~ 15 nucleotides has been mapped in a *magnesium-free environment* with chemical probes [34]. Nucleases digestions performed on dsMCs of various sizes (from 64 to 100 base pairs) and topologies combined with theoretical analyses gave a global picture of the DNA with local unwound structures, which, depending on the size of the circle, may take the form of a small region with opened base pairs or of a sharp kink of the double helix that preserves base pairing [19]. An electro cryo-tomography study reported a conformational heterogeneity of topoisomers of dsMCs, with a distribution of conformations specific of each topoisomer [25]. Molecular dynamics simulations suggested, for a population of topoisomers with a given superhelicity, large fluctuations in the extent of writhe and in the level of compaction. This structural feature might also exist with our smaller topoisomers, and the half-time life of the population of topoisomers with localized denatured regions and unpaired bases of significant length might be too short to be trapped by the Nuclease SI or the DNAse I.

Our binding experiments indicate that in a magnesium-containing buffer, hRPA does bind supercoiled dsMCs giving rise to two nucleoprotein complexes, C_a_ and C_b_. The binding reaction in the presence of magnesium is nevertheless not as efficiently as that that occurred in the absence of magnesium. For instance, at 300 nM hRPA, 80% of the DNA is assembled with hRPA in the absence of magnesium (Fig 5A and 5C) whereas this percentage falls to at most 44% with magnesium (Table 1). A similar behavior has been observed in the case of the interaction between *Ec* SSB proteins and the T_-5_ topoisomer (S4 Fig) and in the case of the interaction of histone f1 with supercoiled DNA [60,61]. These results clearly illustrate that binding properties of proteins in the presence of magnesium cannot be deduced from binding experiments performed in the absence of magnesium. The most abundant dsMC present in the C_a_ and C_b_ nucleoprotein complexes is T_-1_ whereas the most abundant *unbound* dsMC at the reaction equilibrium is the initial substrate (Fig 7, Table 2). Why is the binding of hRPA on T_i_ (i ≤ -2) disfavored? hRPA binds single-stranded DNA in a sequential manner, adopting first a compact (8–10 nucleotides occluded), next an elongated compacted (13–22 nucleotides occluded) and finally an elongated extended conformation (30 nucleotides occluded) [62]. It is possible that the hyper-negative supercoiling, by inducing the formation of a compact structure with a high degree of writhe prevents the sequential binding of hRPA along the DNA and the formation of the stable elongated extended conformation occluding 30 nucleotides. The negatively supercoiled T_-1_ topoisomer may have a unique conformational dynamics that allows hRPA to go through its three sequential binding stages. In contrast, in the absence of magnesium, the higher is the negative supercoiling, the more hRPA bound per dsMC (Fig 5A). Under these conditions, the stabilization of the double helix by counter ions does not exist and the negative supercoiling is mostly absorbed by twist changes (Fig 4C). These ionic and conformational conditions may then favor the assembly of several hRPA with an elongated extended conformation [63].

As expected, the hRPA-dsMCs complexes observed in the absence of magnesium have a lower mobility than naked DNA, and their mobility decreases as the concentration of hRPA increases (Fig 5A). In contrast, the migration profile of the hRPA-dsMCs complexes assembled in the presence of magnesium is unusual from two perspectives. First, our results show that for all topoisomers, the C_a_ complex that forms at a weaker concentration of hRPA than the C_b_ complex, has a reduced mobility compared to the C_b_ complex, and second, both complexes migrate faster than the T_-1_ and T_-2_ topoisomers (Fig 6A). It is known that the wrapping of the DNA double helix around histones changes the topology of the DNA [35]. In the case of the hRPA-dsMCs complexes, it was possible that the binding of hRPA to the dsMCs induced a topology change leading to complexes that had an unusual behavior in terms of mobility in an electric field. However, the results of the “in gel” relaxation assay (Fig 8) exclude the possibility that the binding of hRPA changes the topology of the DNA. It is therefore possible that the unusual migration of the C_a_ and C_b_ hRPA-dsMC complexes with respect to each other and with respect to the T_-1_ and T_-2_ topoisomers stems from the structure itself of the complexes.

## Conclusion

Our experiments demonstrate that hyper-negatively supercoiled dsMCs in a magnesium-containing buffer differ from relaxed dsMCs by subtle, low magnitude and sequence-independent conformational changes. Such conformational changes can either favor binding of proteins as exemplified by Nuclease SI, or in contrast inhibit protein binding as exemplified by the hRPA heterotrimer and *Ec* SSB. In contrast, in the absence of magnesium, negative supercoiling modulates the twist of DNA molecules and favors the binding of single-stranded DNA binding proteins. Importantly, our results show that DNA binding properties of proteins in the presence of magnesium cannot be deduced from binding experiments performed in the absence of magnesium. Finally, the quantitative data obtained from our experiments may be confronted to theoretical models of DNA supercoiling-induced DNA deformation developed by the physicists of the nucleic acids.

## Supporting information

S1 Fig Procedure of preparation of dsMCs. The preparation of dsMCs includes several steps. (1) Radiolabeling of the linear 235 bp-long ds DNA with [gamma -^32^P]-ATP. The * corresponds to the ^32^P label. (2) Ligation at various concentrations of EtBr (from 0 to 30 μg mL^-1^). (3) EtBr extraction, DNA precipitation. (4) PAGE to separate the different species. (5) Cutting of the bands of the gel corresponding to the dsMCs, DNA electro-elution and precipitation. The dsMCs are eluted from the gel that does not contain chloroquine. The position of migration of the dsMCs, the single-stranded linear and double-stranded linear fragments are indicated.
(TIF)

S2 Fig Relative position of the BglII, BamHI and HindIII restriction sites along the dsMC sequence. At the end of the preparation the dsMCs are radiolabeled on both strands. After the double digestion, only two strands (colored in red on the figure) remain radiolabeled. The BglII/ BamHI double digestion generates radiolabeled fragments of 21 and 214 nucleotides after denaturation of the digestion products. The BamHI/HindIII digestion generates radiolabeled fragments of 26 and 209 nucleotides after denaturation of the digestion products.
(TIF)

S3 Fig Sites of structural changes induced by the hyper-negative supercoiling detected by DNAse I. The enzymatic probe used to map the fine structure of the T_0_ (lanes 1, 5, 9 and 14) and T_-5/-6_ (2, 6, 10 and 13) topoisomers is DNAse SI. DNAse I is at 1 μU μL^-1^ and DNA at 0.5 nM. After the DNAse I reaction, the samples are treated to remove the proteins as described in the Materials and Methods section. The DNAs are precipitated and submitted to the BamHI+HindIII (left panel) or BglII+BamHI (right panel) double digestion to only visualize DNA fragments from one of the two radiolabeled strands. The reaction products are analyzed on two different sequencing gels (8% to see long DNA fragments, 12% to see short DNA fragments) as indicated. G and G+A lanes correspond to the products of the Maxam and Gilbert reactions to identify specifically the guanines (G lanes; lanes 4, 8 and 15) or the guanines and adenines (G+A lanes; lanes 3, 7, 11 and 12) in the sequence.
(TIF)

S4 Fig Binding *Ec* SSB to the T_-5_ topoisomers of dsMCs. The T_-5_ topoisomer is at 0.5 nM and the concentration of *Ec* SSB protein ranges from to 0 to 83 ng μL^-1^ (0, 1, 3, 9, 28, 83). The interaction buffer contained, in addition to 25 mM Tris-HCl pH 8, 50 mM NaCl, 1 mM DTT, either 10 mM MgCl_2_ (upper panel) or 1 mM EDTA (lower panel). For both titrations, the samples are analyzed by electrophoresis on native 4% polyacrylamide gels (29:1 = acrylamide:bisacrylamide mass ratio) made in TAcMg (44.5 mM Tris-Base pH 8 (pH adjusted with acetic acid), 10 mM MgCl_2_) when interaction is performed in the presence of Mg^2+^ or TBE 0.5x when interaction is performed in the presence of EDTA. Electrophoresis is performed at 150 V, 4°C and for 4 h.
(TIF)
